# Microbial Antagonism in Food-Enrichment Culture: Inhibition of Shiga Toxin-Producing *Escherichia coli* and *Shigella* Species

**DOI:** 10.3389/fmicb.2022.880043

**Published:** 2022-06-23

**Authors:** Tanis C. McMahon, Cesar Bin Kingombe, Amit Mathews, Karine Seyer, Alex Wong, Burton W. Blais, Catherine D. Carrillo

**Affiliations:** ^1^Research and Development, Ottawa Laboratory (Carling), Ontario Laboratory Network, Canadian Food Inspection Agency, Ottawa, ON, Canada; ^2^Department of Biology, Carleton University, Ottawa, ON, Canada; ^3^Independent Researcher, Ontario, ON, Canada; ^4^Microbiology, Greater Toronto Area Laboratory, Ontario Laboratory Network, Canadian Food Inspection Agency, Toronto, ON, Canada; ^5^Microbiology (Food), St-Hyacinthe Laboratory, Eastern Laboratories Network, Canadian Food Inspection Agency, St-Hyacinthe, QC, Canada

**Keywords:** bacteriocin, bacteriophage, Shiga toxin-producing *Escherichia coli*, *Shigella*, foodborne pathogen

## Abstract

Bacterial pathogens, such as Shiga toxin-producing *Escherichia coli* (STEC) and *Shigella* spp., are important causes of foodborne illness internationally. Recovery of these organisms from foods is critical for food safety investigations to support attribution of illnesses to specific food commodities; however, isolation of bacterial cultures can be challenging. Methods for the isolation of STEC and *Shigella* spp. from foods typically require enrichment to amplify target organisms to detectable levels. Yet, during enrichment, target organisms can be outcompeted by other bacteria in food matrices due to faster growth rates, or through production of antimicrobial agents such as bacteriocins or bacteriophages. The purpose of this study was to evaluate the occurrence of *Shigella* and STEC inhibitors produced by food microbiota. The production of antimicrobial compounds in cell-free extracts from 200 bacterial strains and 332 food-enrichment broths was assessed. Cell-free extracts produced by 23 (11.5%) of the strains tested inhibited growth of at least one of the five *Shigella* and seven STEC indicator strains used in this study. Of the 332 enrichment broths tested, cell-free extracts from 25 (7.5%) samples inhibited growth of at least one of the indicator strains tested. Inhibition was most commonly associated with *E. coli* recovered from meat products. Most of the inhibiting compounds were determined to be proteinaceous (34 of the 48 positive samples, 71%; including 17 strains, 17 foods) based on inactivation by proteolytic enzymes, indicating presence of bacteriocins. The cell-free extracts from 13 samples (27%, eight strains, five foods) were determined to contain bacteriophages based on the observation of plaques in diluted extracts and/or resistance to proteolytic enzymes. These results indicate that the production of inhibitors by food microbiota may be an important challenge for the recovery of foodborne pathogens, particularly for *Shigella sonnei*. The performance of enrichment media for recovery of *Shigella* and STEC could be improved by mitigating the impact of inhibitors produced by food microbiota during the enrichment process.

## Introduction

Foodborne illnesses due to Shiga toxin-producing *Escherichia coli* (STEC) and *Shigella* spp. are an important public health concern around the world (EFSA, 2013; Gould et al., 2013; Thomas et al., 2015; Adam and Pickings, 2016; The et al., 2016). In Canada, it is estimated that there are approximately 1,200 domestically-acquired cases of foodborne shigellosis and approximately 33,000 illnesses attributed to STEC per year (Thomas et al., 2013). Infections by both STEC and *Shigella* can result in serious illnesses. Complications such as hemolytic-uremic syndrome (HUS), hemorrhagic colitis and Reiter’s syndrome can have long-term effects and are sometimes fatal (Donnenberg and Whittam, 2001; Perelle et al., 2004; Warren et al., 2006; Smith et al., 2014; The et al., 2016). Food contamination with *Shigella* is exclusively from human sources (Warren et al., 2006), whereas animals are important reservoirs associated with foodborne STEC (Kim et al., 2020).

Infection with STEC or *Shigella* spp. can occur due to ingestion of as little as 10–100 cells (Kothary and Babu, 2001; Thorpe, 2004). Therefore, methods used to test foods for these pathogens must be very sensitive, and must be able to identify a small number of target cells that are likely present as a miniscule portion of the food microbiota. Current methods used to detect foodborne STEC and *Shigella* in Canada, United States, and Europe involve an enrichment step followed by screening of enrichment cultures for characteristic virulence genes (Bin Kingombe et al., 2006; Warren et al., 2006; ISO, 2012; Blais et al., 2014; USDA-FSIS, 2019). Typically, an enrichment procedure is conducted with the intention of favoring the growth of the target bacteria while limiting the growth of non-target or background bacteria present in the sample. Nonetheless, the non-target bacteria can often outcompete the target pathogen under these conditions due to faster growth rates, or production of compounds that actively interfere with their growth (Uyttendaele et al., 2001; Blais et al., 2019). This can lead to false-negative results in cases where the pathogen dies, or is present at a proportionally lower level after enrichment, and cannot be detected or isolated in downstream analyses (Kozak et al., 2013). There are many examples of foodborne outbreaks associated with STEC or *Shigella* spp. in which pathogens were not successfully recovered from implicated foods despite strong epidemiological evidence supporting food attribution (Heier et al., 2009; Lewis et al., 2009; Marshall et al., 2020; Mikhail et al., 2021). Failure to recover target pathogens may result in delays in attribution of an outbreak to a food commodity.

A number of studies have investigated growth dynamics in food enrichment cultures to gain a better understanding of the growth of interfering organisms in these environments (Jarvis et al., 2015; Margot et al., 2016; Ottesen et al., 2016; Kang et al., 2021). The aim of many of these studies has largely been to catalog species in the food microbiome that can interfere with the successful recovery of target pathogens in enrichment cultures, and to evaluate the strengths and weaknesses of different methodological approaches in reducing the growth of non-target organisms. Few studies have looked specifically at the role of antimicrobial compounds produced by food microbiota in food enrichment culture. Although, one study of tomato enrichment culture microbiomes identified *Paenibacillus* as a potential *Salmonella*-inhibiting organism in this system, based on known activity of this organism (Ottesen et al., 2013).

Bacteria can produce a variety of antimicrobial compounds. Almost all bacteria encode bacteriocins, which are antimicrobial peptides (Kim et al., 2014; Yang et al., 2014; Simons et al., 2020). Bacteriocins produced by Gram-negative bacteria that target related species are classified as colicins (25–80 kDa) or microcins (10 kDa; Duquesne et al., 2007; Yang et al., 2014; Mader et al., 2015). These bacteriocins damage host cells through pore formation, DNA/RNA degradation, protein synthesis inhibition or DNA replication inhibition (Alonso et al., 2000; Duquesne et al., 2007; Yang et al., 2014). Another mechanism of microbial antagonism is through the production of bacteriophages that can infect and kill bacteria (Penadés et al., 2015; Shahin et al., 2019). Bacteriophages have two cycles of viral reproduction: the lysogenic and lytic cycles. In the lysogenic cycle, the bacteriophage genome is integrated into the bacterial host’s genome as a prophage and is propagated through replication of the host’s chromosome without damaging the host cell (Parasion et al., 2014; Penadés et al., 2015). Induction involves conversion of the lysogenic infection into a lytic infection, where the host’s machinery is used to produce mature phages, which are released through lysis of the infected cells. Bacteriophages in the lysogenic cycle can be induced into the lytic cycle when the bacteria undergo stress or through induction of the SOS response. Most phages can only affect a subset of bacteria within a species, and specificity of the phage depends on the receptors to which it binds (Koskella and Meaden, 2013; Parasion et al., 2014). Finally, other antibiotic compounds (e.g., lipopeptides, aminoglycosides, tetracyclines, and aminocoumarins) may be produced by bacterial groups such as Actinomycetales, Bacillales, and Enterobacterales (Mandal et al., 2013; Challinor and Bode, 2015; Mohr, 2016).

The purpose of this study was to evaluate the occurrence of antimicrobial compounds produced by food microbiota in food enrichment cultures. *Shigella* spp. and STEC were selected for this study as they are related genera that have low infectious doses, and are among the most difficult pathogens to recover from foods. Cell-free extracts derived from food-associated bacterial strains and from food enrichments [modified Tryptone Soya Broth (mTSB) or *Shigella* broth (SB)] were tested for inhibitory activity against STEC and *Shigella*. Samples containing inhibitors were further characterized to determine the likely mechanisms of inhibition. Results of this study will be of great value in the development of improved methods for a more reliable recovery of STEC and *Shigella* spp. from foods.

## Materials and Methods

### Growth and Maintenance of Bacterial Strains

A selection of 200 predominantly Enterobacteriaceae strains, most of which were previously isolated from food enrichment broths, were selected for this study (Supplementary Table S1). Seven *E. coli* strains representing clinically important STEC serotypes and five *Shigella* strains were used as the indicator strains for testing sensitivity to inhibition from cell-free extracts (Table 1). All strains were stored at −80°C in 15% glycerol and were plated on Brain-Heart Infusion agar (BHI; OXOID, Nepean, ON, Canada) overnight (14–16 h) at 37°C prior to use.

**Table 1:** STEC and *Shigella* strains used as indicator organisms.

Isolate	Genus	Species	Serotype	Description/Isolation source
OLC0024	*Shigella*	*sonnei*		ATCC29930/feces
OLC2340	*Shigella*	*sonnei*		Pasta salad outbreak/human feces
OLC0603	*Shigella*	*flexneri*	1a	ATCC25929/human feces
OLC1597	*Shigella*	*flexneri*	1b	ATCC12022/missing
OLC0608	*Shigella*	*dysenteriae*		Human feces
OLC0455	*Escherichia*	*coli*	O111:H11	STEC *stx1a*, *eae*/missing
OLC0464	*Escherichia*	*coli*	O26:H11	STEC *stx1a*, *eae*/missing
OLC0675	*Escherichia*	*coli*	O145:NM	STEC *stx1a*, *eae*/human feces
OLC0679	*Escherichia*	*coli*	O103:H2	STEC *stx1a*, *eae*/human feces
OLC0710	*Escherichia*	*coli*	O121:H19	STEC *stx2a*, eae/human feces
OLC0716	*Escherichia*	*coli*	O45:H2	STEC *stx1a*, *eae*/human feces
OLC0797	*Escherichia*	*coli*	O157:H7	STEC *stx1a*, *sxt2a*, *eae*/human feces

### Preparation of Cell-Free Extracts

Cell-free extracts were prepared from (1) overnight cultures of bacterial strains, and (2) food enrichment cultures from negative samples collected by the CFIA’s food testing laboratories in Ottawa (ON), Toronto (ON), and Saint-Hyacinthe (QC).

**Figure 1:**
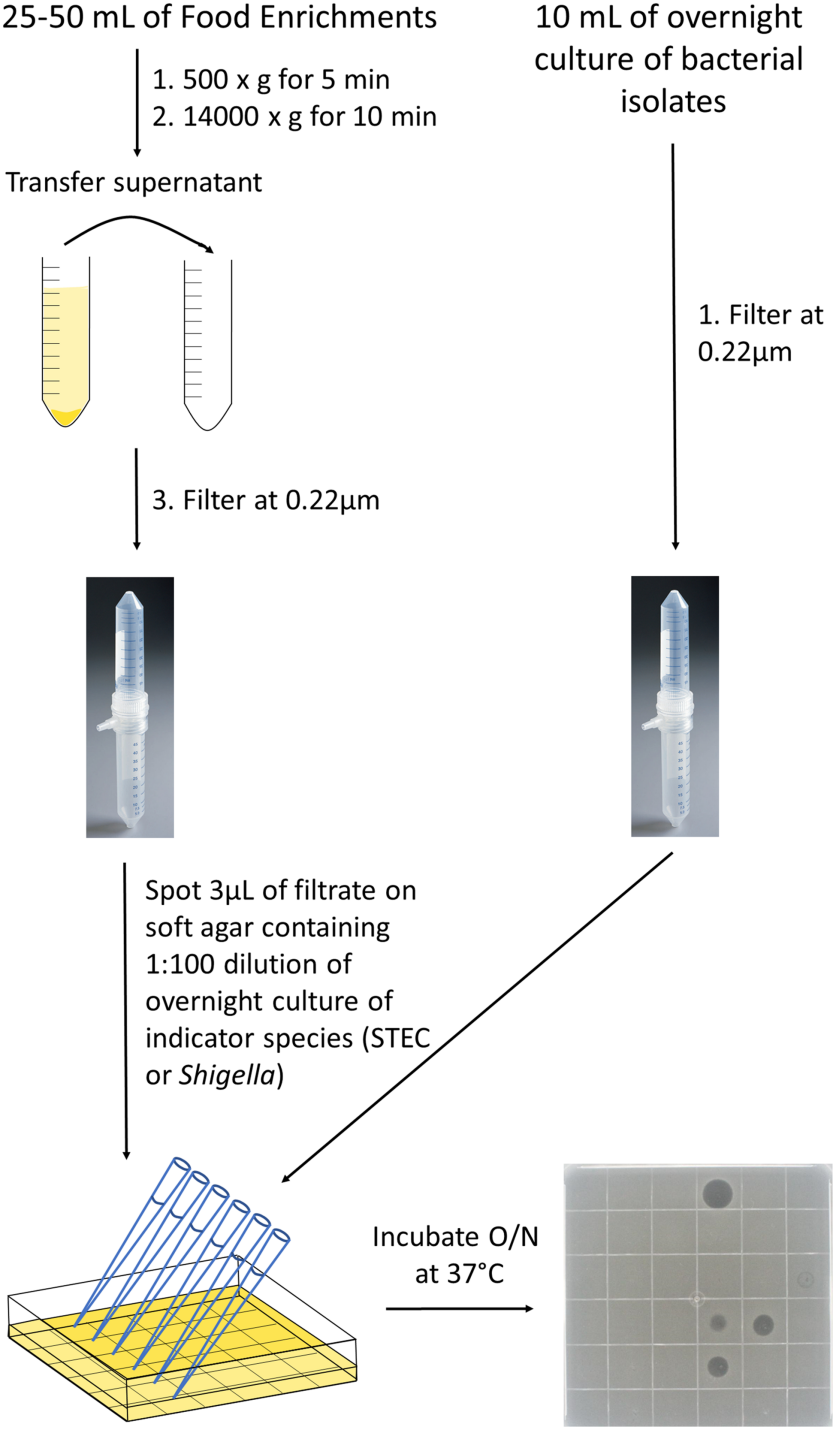
Detection of inhibitors in cell-free extracts from food enrichments or bacterial strains. Bacterial strains were grown overnight in 10 ml of Nutrient broth at 37°C (right side) and food enrichments were incubated according to MLFP-26 or MLFP-30 methods. For food enrichments (left side), an aliquot of 25–50 ml was taken and centrifuged first at 500 × *g* to remove large food particles and then at 14,000 × *g* to remove bacterial cells and debris. Supernatants from cell enrichments or overnight bacterial cultures were then filtered through a 0.22 μm vacuum filter to remove remaining food particles and bacterial cells. Filtrates were spotted on soft agar containing indicator strains. Filtrate spots were absorbed into the agar, and plates were incubated overnight at 37°C. The following day, pictures were taken to observe the clearings and the diameter of the clearings were measured in millimeters. A representative plate with 35 different cell-free extracts and a negative control spotted in a 6 × 6 grid is shown.

Bacterial strains (*n* = 200; Supplementary Table S1) were grown overnight at 37°C in 10 ml of Nutrient broth (OXOID) and broths were filtered using a 0.22 μm Vacuum filter (EMD Millipore Steriflip™ Sterile Disposable Vacuum Filter Units; Thermo Fisher Scientific, Ottawa). The cell-free extracts were stored at 4°C for up to 3 months and at −20°C for longer storage (Figure 1).

Food products were sampled between the fall 2016 and winter 2017 and were representative of the types of foods tested in regulatory food testing programs (Supplementary Table S2). Extracts were prepared from 332 enrichment cultures derived from 235 food samples. Categories of food products tested included fruits [46 (14%)], salads and coleslaws [78 (23%)], meats [85 (26%)] and vegetables, cheese and other [123 (37%)]. Enrichment cultures were generated using methods described in the Canadian Compendium of Analytical Methods: either the method for detection of *Shigella* spp. in foods (MFLP-26; *n* = 23; Bin Kingombe et al., 2006) or the method for detection of STEC in foods (MFLP-30; *n* = 115; Microbiological Methods Committee, 2012) or both (*n* = 97). The meats and cheeses were only enriched in mTSB and the rest were enriched in SB or both broths. For the MFLP-26 method, samples are enriched in a 1:10 dilution of sample to *Shigella* broth (SB; SB base, OXOID) with Tween-80 (Sigma, Markham, ON, Canada) containing 0.5 μg/ml of novobiocin (Sigma-Aldrich, Oakville, ON). The SB is enriched for 20 h at 42°C in a CO_2_ incubator or CO_2_ jar system. The MFLP-30 method involves a 1:10 dilution of sample to modified Tryptone Soya Broth (mTSB, OXOID) containing 20 μg/ml of novobiocin, followed by aerobic incubation of the enrichment at 42°C for 18–24 h. Typically, 25 g of food are enriched in 225 ml of enrichment broth using both methods. Note that enrichments were negative for targeted pathogens and that bacterial growth was observed for all samples based on turbidity of broths.

Enrichment broths were stored at 4°C prior to use. Cell-free extracts were prepared from 25 to 50 ml of enrichment broth by centrifugation at 500 × *g* for 5 min, transferring the supernatant to a new tube, followed by centrifugation at 14000 × *g* for 10 min (Figure 1). The supernatant was then filtered as described above and stored at 4°C or −20°C. The pellets from the high-speed spin were resuspended in 30% glycerol and stored at −80°C.

### Detection of Inhibitors in Cell-Free Extracts

The methods for evaluating the inhibitory activity of cell-free extracts were modified from methods developed by (Arici et al., 2004) and (Vijayakumar and Muriana, 2015; Figure 1). *Shigella* and STEC were grown overnight in 10 ml of Nutrient Broth (NB, OXOID) at 37°C to obtain a concentration of approximately 10^8^ cells/ml. Overnight cultures of each of the indicator strains were enumerated to assess reproducibility of this approximation for initial experiments, but enumeration was not done routinely (data not shown). The overnight *Shigella* or STEC culture was added at a concentration of approximately 1 × 10^6^ cells/ml into soft agar cooled to 40°C [NB (OXOID) containing 0.5% (w/v) bacteriological agar (Sigma-Aldrich)] and poured into a Petri dish. Once the plates were solidified, 3 μl spots of the cell-free extracts were added and left to absorb into the agar before incubating plates overnight at 37°C. To enable high-throughput analyses, multichannel pipets were used to generate 36 spots on square Petri plates. All extracts were tested in triplicate.

### Isolation of Bacteria Producing Inhibitors From Food Enrichments

The method from Henning et al. (2015) was used to isolate the inhibitor-producing bacteria from a subset of 13 of the food enrichment broths that were active against at least one indicator organism. Dilutions of the enrichment broths were spread plated on nutrient agar (OXOID) then immediately overlaid with a thin nutrient agar sandwich layer. The plates were incubated at 37°C overnight before a soft agar containing ~1 × 10^6^ cells/ml of the indicator species (*Shigella sonnei* OLC2340) was layered on top (see above). The triple layer agar was incubated overnight at 37°C. Note that only *S. sonnei* OLC2340 was used in this experiment, due to the complexity of the method, and the susceptibility of this strain to most of the cell-free extracts.

Following incubation, the triple agar plates were inverted and placed onto their Petri dish covers, and colonies surrounded by zones of clearing were streaked onto a new nutrient agar plate. The streaked plates were incubated overnight at 37°C and isolated colonies were patched onto two nutrient agar plates. The two plates were incubated at 37°C for 4 h and then one of the duplicate plates was overlaid with soft agar containing ~1 × 10^6^ cells/ml of *S. sonnei* OLC2340. The plates were again incubated at 37°C overnight. Isolates recovered from these plates were streaked for purity, then re-tested to confirm inhibitory activity. Isolates confirmed to cause inhibition were stored and maintained as described above.

### Testing Cell-Free Extracts for Proteinaceous Properties, Bacteriophage, and pH

Proteolytic enzymes were used to assess the proteinaceous nature of the inhibitory cell-free extracts (Elayaraja et al., 2014). Proteinase K (Thermo Fisher Scientific) and trypsin (Sigma-Aldrich) were added to the extracts at a final concentration of 1 mg/ml or 1X, respectively. The extracts were incubated for 2 h at 30°C before spotting on soft agar containing target bacteria as described above, alongside an untreated control. The presence of bacteriophage was assessed using the dilution method from Hockett and Baltrus (2017). Cell-free extracts were serially diluted two times in nutrient broth (1:10 and 1:100). Dilutions were spotted on soft agar as described above. Bacteriophage presence was confirmed if individual plaques were visible in the diluted samples and/or the clearing was unaffected by proteolytic enzymes. The pH of the cell-free extracts were measured with pH indicator strips (Thermo Fisher Scientific).

## Results

### Inhibition of Growth of *Shigella* and STEC by Cell-Free Extracts Derived From Bacterial Strains

**Figure 2:**
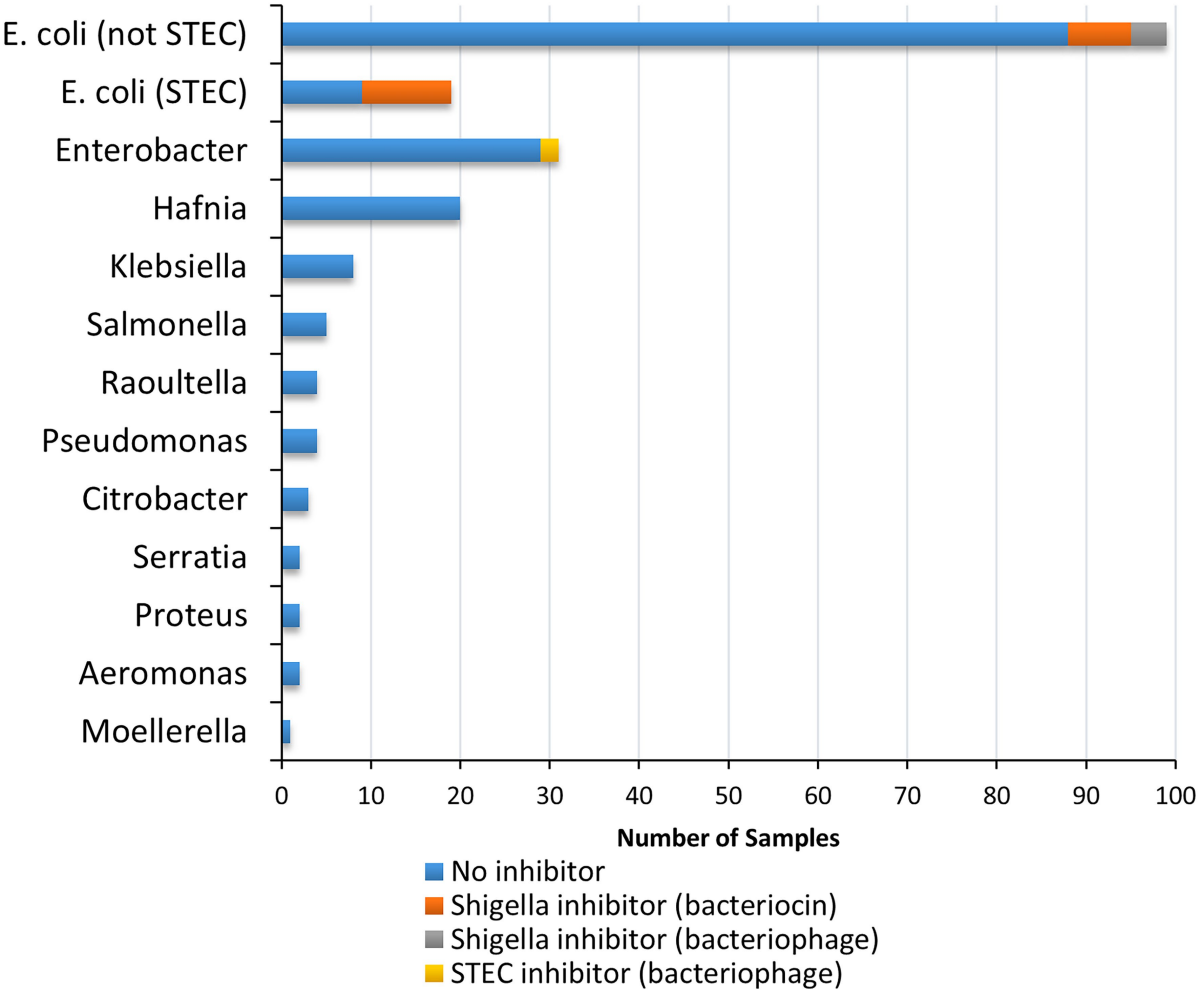
Species and inhibitory activity of foodborne bacterial strains tested against *Shigella* and STEC. In total cell-free extracts from 200 bacterial strains were tested on five *Shigella* spp. strains (two *Shigella sonnei*, two *Shigella* flexneri, and one *Shigella dysenteriae*) and seven STEC (Serotypes O26, O45, O103, O111, O121, O145, and O157) samples. Relative proportion of strains producing inhibitors are indicated according to genus impacted (*Shigella* spp. vs. STEC), and predicted inhibitor (bacteriocin vs. bacteriophage; see legend).

**Figure 3:**
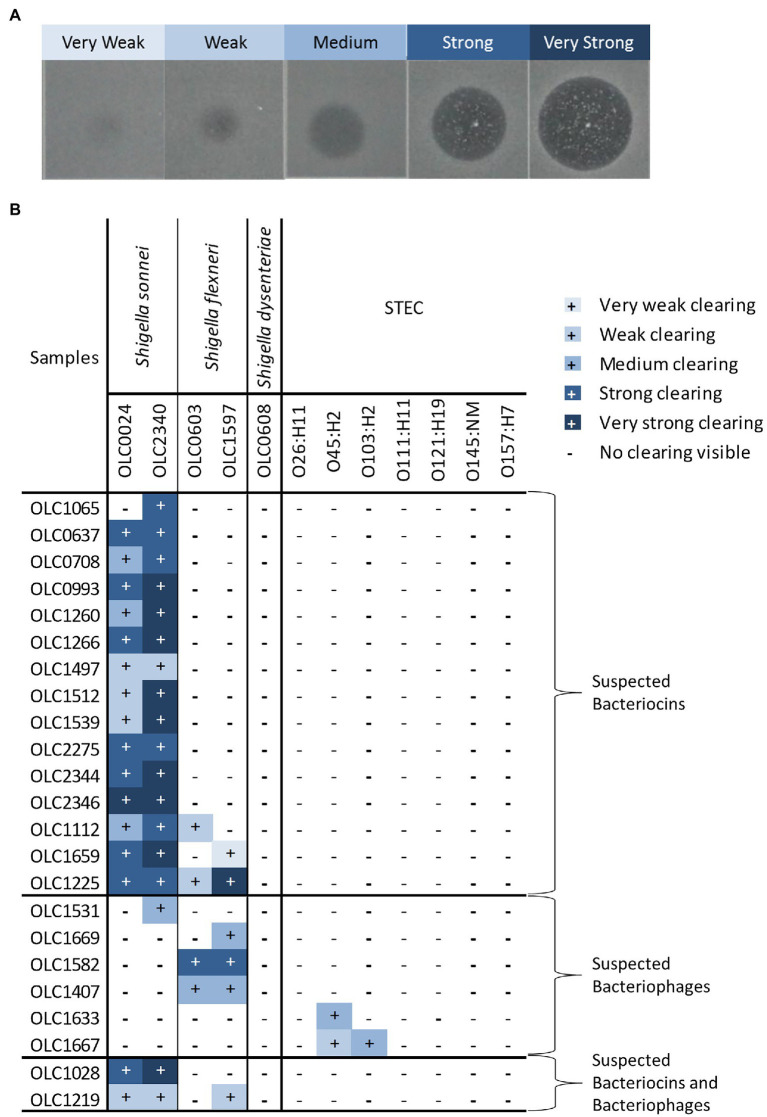
Strengths of inhibitory activity of bacterial strains. **(A)** Representative image of the different strengths of the inhibitory activity observed in the strains used in this study. Level of activity (e.g., very weak to very strong) was classified based on diameter of the zone of clearing and opacity of the spot. **(B)** Relative strength of inhibitory activity of cell-free extracts from bacterial strains on *Shigella* spp. and STEC is indicated. The strains are grouped based on predicted inhibitor present in the sample (bacteriocins, bacteriophage, or both).

Cell-free extracts from 200 bacterial strains, primarily recovered from food (Supplementary Table S1), were tested to detect inhibition of growth of five strains of *Shigella* and seven strains of STEC (Table 1). Twenty-three of the 200 strains tested (11.5%) inhibited the growth of at least one of the 12 indicator organisms (*Shigella* and EHEC) used in this study. Most of the strains tested were Enterobacteriaceae (194/200), except for six strains (Pseudomonadaceae and Aeromonadaceae; Figure 2; Supplementary Table S1). The three main genera evaluated were *Escherichia*, *Enterobacter* and *Hafnia* [118 (48.5%), 31(14.5%), and 20(10%), respectively]. Cell-free extracts produced by 21 *E. coli* (10.5% of all strains, 17.8% of *E. coli*) inhibited growth of at least one strain of *Shigella*, and cell-free extracts from two *Enterobacter* spp. strains (1% of all strains, 6.5% of *Enterobacter* spp.) inhibited growth of at least one strain of STEC (Figure 2). Most of the inhibitor-producing *E. coli* affected *S. sonnei* (18 of 21, 86%), with a smaller proportion (*n* = 7, 33%) affecting *Shigella flexneri*. *E. coli* classified as STEC were more likely to produce bacteriocins (10 of 19 strains, 52.6%), compared to other *E. coli* (11 of 99 strains, 11.1%). In most samples, both strains of *S. sonnei* (OLC0024 and OLC2340) were inhibited (16 out of 18) whereas only three out of the seven extracts affecting *S. flexneri* inhibited both of the strains used in this study (Figure 3). The inhibitor-producing *Enterobacter* spp. affected *E. coli* O45 (two extracts) and *E. coli* O103 (one extract; Figure 3). None of the strains evaluated in this study inhibited growth of *Shigella dysenteriae*.

The relative strength of inhibition varied among cell-free extracts and was assessed based on the diameter of the zone of inhibition and the opacity of the clearing (Figure 3A). Samples were designated as very weak (3 mm diameter) to very strong (11 mm diameter; Figure 3; Supplementary Table S3). The inhibitory activity of cell-free extracts from *E. coli* on *S. sonnei* was generally categorized as strong and very strong, whereas inhibitory activity on *S. flexneri* was largely determined to be medium or weak. Similarly, the cell-free extracts from *Enterobacter* spp. that inhibited STEC produced medium or weak inhibition of growth.

### Inhibition of *Shigella* and STEC From Cell-Free Extracts From Food Enrichments

**Figure 4:**
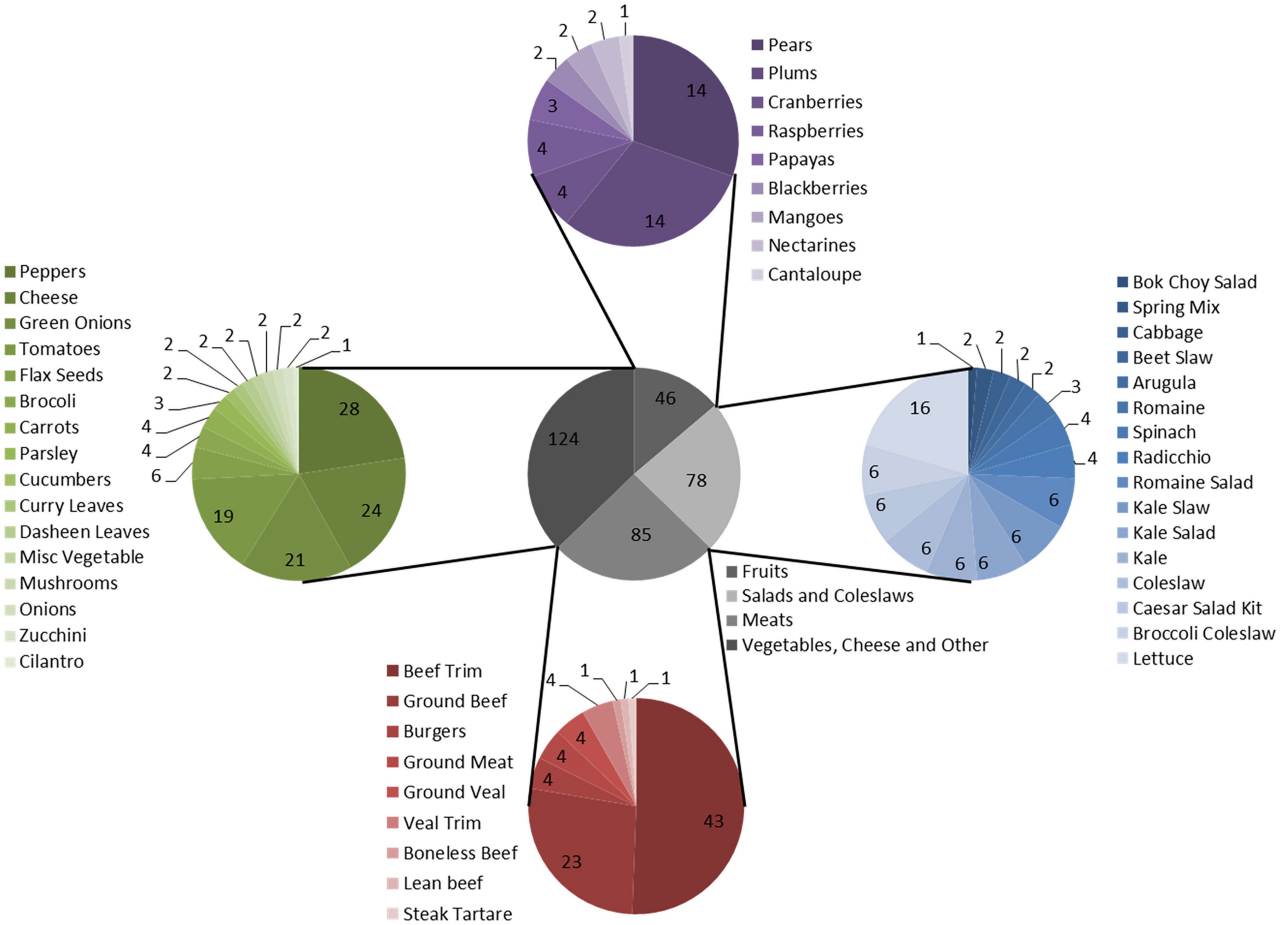
Food product enrichment cultures tested for production of inhibitors. Gray pie chart: Categories. Blue pie chart: Salads and Coleslaws. Red pie chart: Meats. Green pie chart: Vegetables, Cheese, and Other. Purple pie chart: Fruits. Total food enrichment samples tested was 332 samples.

**Figure 5:**
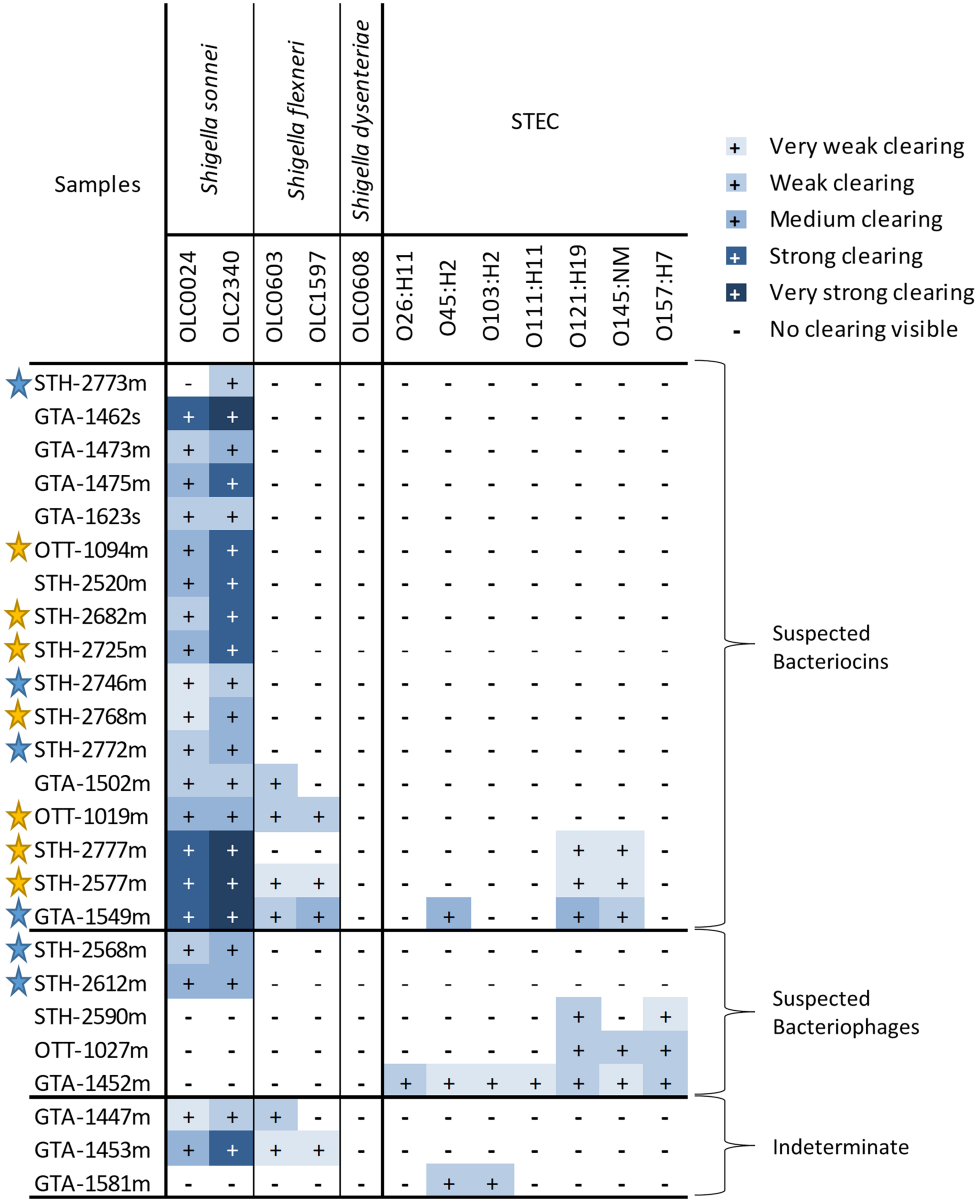
Strengths of inhibitory activity of food enrichments on *Shigella* and STEC. Relative strength of inhibitory activity of extracts from mTSB (“m” in sample name) and SB enrichment (“s” in sample name) on *Shigella* spp. and STEC is indicated (see Figure 3A for representative images of different strengths of inhibitory activity). The enrichments are grouped based on predicted inhibitor present in the sample (bacteriocins, bacteriophage, or both). Samples were deemed to be indeterminate if observed inhibition of untreated samples was weak and impacts of proteolytic enzymes and dilutions could not be observed. Stars indicate samples for which attempts were made to recover isolates of the bacteria responsible for inhibition. Yellow stars indicate that an isolate producing an inhibitor was recovered, blue stars indicate attempts to recover isolates from samples were unsuccessful.

Cell-free extracts from 332 food-enrichment broths derived from 235 food products (Figure 4; Supplementary Table S2) were evaluated to determine prevalence of inhibitors to *Shigella* spp. or STEC in food enrichments. Of the 332 enrichment broths tested, cell-free extracts from 25 (7.55%) samples inhibited growth of at least one of the 12 *Shigella* or STEC strains used in this study (Figure 5). Twenty-one samples (6%) inhibited growth of *Shigella* spp. and seven (2%) inhibited growth of STEC (Figure 5). Among the 25 cell-free extracts containing inhibitors, 21 (84%) affected *S. sonnei*, six (24%) affected *S. flexneri* and seven (28%) affected STEC growth (Figure 5). One of the extracts (GTA-1452) inhibited growth of all STEC strains tested in this study and three extracts inhibited both *Shigella* and STEC strains. None of the extracts affected growth of *S. dysenteriae*.

As with the extracts from bacterial strains, the relative strength of inhibition was assessed based on the diameter of the zone of inhibition and the opacity of the clearing (Figure 3A). Strength of inhibition varied among the extracts and among indicator organisms tested (Figure 5; Supplementary Table S3). Inhibitory activity against *S. sonnei* tended to be strong, whereas inhibitory activity against *S. flexneri* and STEC was weaker (Figure 5). *Shigella* inhibitors were found in two (1.7%) of the 120 SB extracts (curry leaves and broccoli slaw) and STEC inhibitors were present in seven (3.2%) of 213 mTSB extracts tested. In contrast, mTSB extracts examined in this study were more likely to contain *Shigella* spp. inhibitors (8.9%), whereas none of the SB extracts contained STEC inhibitors. All the food enrichments had relatively neutral pH values between 6.0 and 7.5.

**Figure 6:**
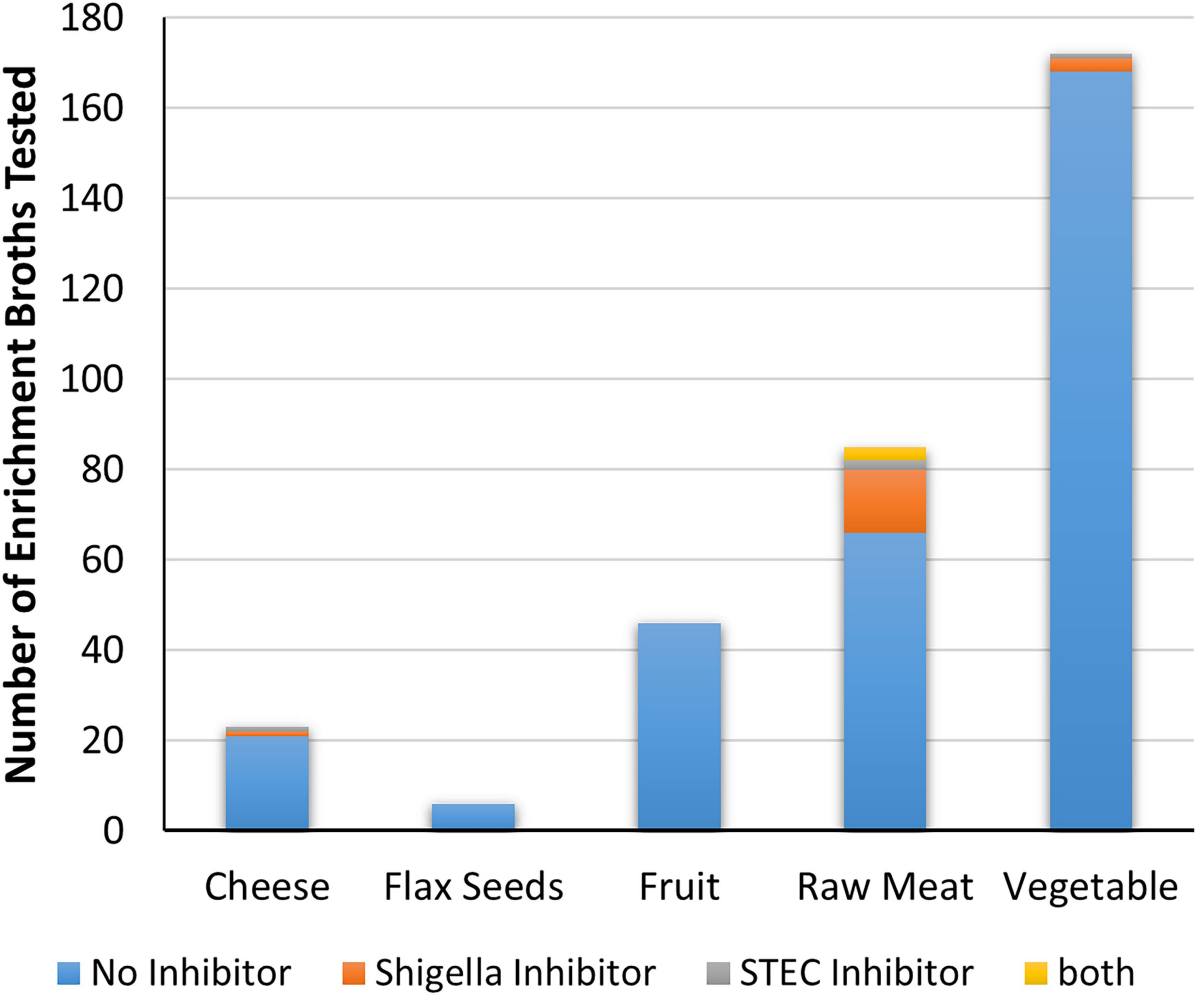
Inhibitory activity of food enrichments on *Shigella* and STEC. The number of enrichment broths from each food category that did not produce inhibitors, or are inhibitory to either *Shigella*, STEC or both are indicated.

Cell-free extracts derived from the 85 meat mTSB extracts were most likely to contain inhibitors to either *Shigella* and/or STEC (19 extracts, 22% of meat enrichments; Figure 6; Supplementary Table S2). These extracts inhibited growth of *Shigella* spp. (14 extracts), STEC (two extracts) or both (three extracts). Two of the 23 cell-free extracts derived from cheese mTSB enrichments inhibited growth of either *Shigella* spp. or STEC. In contrast, only four of the 224 extracts derived from plant products (flax seeds, fruit, and vegetables) enriched in mTSB (*n* = 104) or SB (*n* = 120) contained inhibitors. In the 97 samples where plant products were enriched in both mTSB and SB, inhibition was only observed in one of the broths. For two of these samples (curry leaves and broccoli slaw), inhibitory compounds were detected in the SB extracts, but not the mTSB extracts. For two samples (both leafy greens) inhibitory compounds were detected the mTSB extracts, but not in the SB extracts.

### Characterization of Inhibitory Properties of Cell-Free Extracts

**Figure 7:**
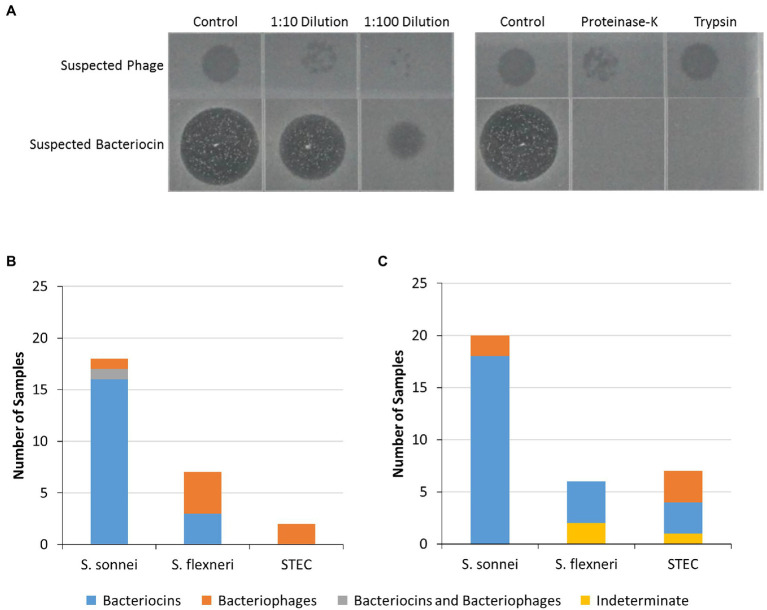
Characterization of inhibitors in isolate and food-enrichment cell-free extracts. Cell-free extracts from food strains and enrichments which inhibited growth of at least one of the indicator strains tested were further evaluated to characterize inhibitors present. **(A)** The cell-free extracts were treated with two proteolytic enzymes (proteinase-K or trypsin) or were diluted (1:10 and 1:100) then spotted on soft agar containing indicator strains. Loss of activity following protein digestion indicated that growth inhibition was likely due to presence of bacteriocins (suspected bacteriocins bottom row), and resilience to protein digestion along with presence of plaques in diluted extracts indicated bacteriophage (suspected bacteriophage, top row). **(B,C)** Inhibitory activity of bacterial isolate extracts **(B)** or food enrichment extracts **(C)** against indicator organisms. Predicted bacteriocins are indicated by blue bars, bacteriophages (orange bars) or both (gray bars). Indeterminate samples could not be assessed due to weak zones of clearing (yellow bars).

All the cell-free extracts causing inhibition in at least one of the indicator *Shigella* or STEC strains were treated with proteolytic enzymes (proteinase-K and trypsin) to determine if inhibition was eliminated by the removal of the protein components of the extracts indicating that inhibitor was likely to be a bacteriocin (Figure 7A). The inhibitory activity of the cell-free extracts from most of the food strains [17 (74%)] and the food enrichments [15 (60%)] were affected by at least one proteolytic enzyme (suspected bacteriocins in Figures 3, 5). For the food enrichment broths, there were two samples that were indeterminate for all inhibited strains and one sample indeterminate for *S. flexneri* due to lack of inhibition in the untreated control. Inconclusive results were likely associated with prolonged storage of these extracts as inhibitory activity was found to generally decrease over time during storage at 4°C (data not shown). For the cell-free extract from OLC1219, the proteinase treatment reduced inhibition for *S. sonnei* strains but not for *S. flexneri* (Figure 3) indicating the presence of different inhibition mechanisms for these two species. Most of the cell-free extracts that inhibited *Shigella* (particularly *S. sonnei*) were affected by proteolytic enzymes (suspected bacteriocins in Figures 3, 5). Cell-free extracts that inhibited STEC were generally not affected by proteolytic enzymes (Figures 3, 5).

To identify bacteriophages, the inhibitory cell-free extracts from the food strains and food enrichments that were resistant to proteolytic digestion were also diluted to assess the presence of plaques (Figure 7A). Plaques were observed in dilutions derived from eight of the cell-free extracts from bacterial strains (suspected bacteriophage in Figures 3, 7B). For strain OLC1219 plaques were observed with *S. flexneri* but not *S. sonnei* indicating that inhibition of *S. flexneri* growth was likely due to bacteriophage, and inhibition against *S. sonnei* growth was likely due to production of bacteriocins (see above). Extracts from the OLC1028 strain were not completely affected by proteolytic enzymes and plaques were observed following treatment with proteolytic enzymes indicating production of both bacteriocin and bacteriophage inhibitors of *S. sonnei*. Plaques were observed in extracts from five of the food enrichment broths (bacteriophage in Figure 5, 7C).

### Recovery of Bacteria Producing Inhibitory Compounds From Food Enrichment Broths

Attempts were made to recover isolates of bacteria producing inhibitors from 13 of the mTSB enrichment broths producing inhibitory compounds (Figure 5, stars) using the triple overlay method (Henning et al., 2015). Inhibiting organisms were successfully recovered from seven of these samples (Figure 5, yellow stars and Table 2). All recovered isolates were determined to be *E. coli* based on whole-genome sequence analysis (data not shown). Cell-free extracts from isolates generally exhibited similar inhibitory properties to the cell-free extracts from enrichment cultures (Table 2; Figure 5, yellow stars). For example, all seven isolates inhibited growth of both strains of *S. sonnei*. Activity of the extracts from six of the *E. coli* isolates was affected by proteolytic enzymes indicating that inhibition was likely due to the production of bacteriocins (Table 2). The isolate recovered from STH-2768 m produced an inhibitor that affected both *S. sonnei* and *S. flexneri*, and was determined to produce bacteriophage as the inhibition was not impacted by proteolytic enzymes.

**Table 2:** Inhibitor-producing organisms recovered from food enrichment cultures.

Strain	Enrichment culture	Serotype	Activity	Species inhibited
OLC3028	STH-2577	O174:H8	Bacteriocin	*Shigella sonnei*
OLC3029	STH-2682	O23:H9	Bacteriocin	*Shigella sonnei*
OLC3030	STH-2725	O123:H16	Bacteriocin	*Shigella sonnei*
OLC3032	STH-2768	O105:H7	Bacteriophage	*Shigella sonnei* *Shigella flexneri*
OLC3035	STH-2777	O153/O178:H11	Bacteriocin	*Shigella sonnei*
OLC3094	OTT-1094	O81:H7	Bacteriocin	*Shigella sonnei*
OLC3095	OTT-1019	O51:H10	Bacteriocin	*Shigella sonnei*

## Discussion

Recovery of the *Shigella* spp. and STEC from foods is important to support food safety investigations and to ensure the timely recall of implicated foods. Yet, this can be extremely challenging, in part due to problems culturing pathogens to detectable levels relative to non-target organisms. Studies conducted using current microbiological methods may underestimate the prevalence of *Shigella* spp. as this organism is easily outcompeted by other Enterobacteriaceae (Fishbein et al., 1971; Pollock and Dahlgren, 1974; Uyttendaele et al., 2001). Similarly, detection of STEC using existing methodology can be difficult due to lack of selective enrichment media, and competition with non-target organisms during food enrichment culture (Duffy et al., 1999; Catarame et al., 2003; Vimont et al., 2006; Knowles et al., 2016; Verhaegen et al., 2016; Blais et al., 2019). Very few studies have examined the impact of antimicrobial compounds produced by food microbiota on pathogen detection. This study was performed to determine whether antimicrobial compounds impacting STEC, or *Shigella* spp. were commonly produced by the microbiota associated with various foods.

### Inhibition of Target Foodborne STEC and *Shigella* spp. by Food Microbiota

A collection of 200 food-associated bacterial strains (Supplementary Table S1) was tested for the production of inhibitory activity against a panel of seven STEC and five *Shigella* strains. The panel of indicator strains was selected to represent clinically important species and strains of pathogens that are targeted in food testing programs. The Gram-negative foodborne bacteria included in this study would typically be highly represented in food-enrichment cultures aimed at recovery of *Shigella*/STEC. Most methods integrate antibiotics such as novobiocin to reduce growth of Gram-positive bacteria, but generally do not include selective agents that reduce growth of Gram-negative bacteria (Vimont et al., 2007). Only two of the 36 *Enterobacter* strains tested inhibited growth of STEC (Figure 3B; OLC1633 and OLC1667). In previous investigations organisms such as *Clostridium* spp., *E. coli*, *Hafnia alvei*, *Brochothrix thermosphacta*, and *Pediococcus acidilactici* present in meats were shown to outcompete STEC; however, the mechanism for this was not identified (Duffy et al., 1999; Kang et al., 2021). Similarly, Paquette et al. (2018) identified *E. coli* strains capable of inhibiting STEC due to production of diffusible antimicrobial compounds (Paquette et al., 2018). While *E. coli* with this activity was not found in the present study, analysis of a larger number of strains or food samples may have led to the identification of strains with similar properties. In contrast, extracts from 20 of the 118 *E. coli* strains tested were found to inhibit growth of *Shigella* spp., particularly *S. sonnei*, indicating that this species is highly susceptible to antimicrobial compounds produced by food microbiota. The inhibition of *Shigella* spp. by antibiotics produced by *E. coli* recovered from human sources was first described over 70 years ago (Halbert, 1948; Halbert and Gravatt, 1949), and *S. sonnei* strains have been used as indicator organisms for bacteriocins detection due to known susceptibility to a variety of colicins (Šmarda and Obdržálek, 2001; Micenková et al., 2014). It is interesting to note that STEC strains were more likely to produce inhibitors impacting growth of *Shigella* spp. compared to non-pathogenic strains. The association of bacteriocins and virulence factors in *E. coli* has been previously reported (Bradley et al., 1991; Micenková et al., 2014). Both of these traits may provide a competitive advantage in certain environments.

Analysis of cell-free extracts derived from a variety of food enrichments was done to evaluate the production of inhibitors by more complex communities of representative food microbiota. While *Shigella* broth would not be used for detection of STEC, and mTSB would not be used for detection of *Shigella*, similar analyses conducted with both types of enrichment broths enabled comparison of inhibitor production by organisms growing in the two media. Inhibition of at least one of the 12 indicator organisms tested was observed for 7.5% of the enrichment cultures tested, making it a relatively common occurrence overall. Inhibitors were more commonly associated with raw meat products enriched in mTSB (22% of raw meat enrichments) relative to plant products (1.8%). Similarly, a recent study found pathogen-killing bacteriophage to be more prevalent in raw beef and chicken than in vegetables and seafood (Premarathne et al., 2017). The relatively high prevalence of *Shigella* inhibition in the mTSB enrichments may be due to the species of bacteria present in the food matrix rather than differences in the enrichment broths. For example, *E. coli* is known to be associated with animal products (Barco et al., 2015). *Shigella*-inhibiting *E. coli* has long been known to be associated with human clinical samples (Halbert, 1948; Halbert and Gravatt, 1949; Levine and Tanimoto, 1954), but to confirm animal association, samples of animal fecal matter should be further examined. The association of inhibition to mTSB is partly due to the fact that meat and cheese products were not enriched in *Shigella* broth in this study. Paired enrichments of raw meat products in both broths could be evaluated to confirm the association of inhibitor-production with raw meats rather than enrichment broths. In the 97 plant products enriched with both broths, no association of the inhibiting organisms to the enrichment broths was observed; however, only four of the 224 broths examined produced inhibitors.

Similar to what was observed with the bacterial strains, *S. sonnei* was the indicator species that was the most sensitive to inhibition (21 out of 25 extracts), followed by *S. flexneri* and STEC (Figure 5). The observation of similar trends between the bacterial strains and enrichments indicates that the bacterial strains used in this study are representative of the bacteria in food enrichments. Most enrichments affected only one species (e.g., *S. sonnei* or STEC for 19 of 25 extracts) however there were some samples that showed a broader activity. For instance, cell-free extracts from one enrichment culture inhibited all seven STEC strains (GTA-1452, Figure 5). There were also three enrichment broths that inhibited both STEC and *Shigella* strains (GTA-1549, STH-2577 and STH-2777). This is likely indicative of an inhibitor that has a larger spectrum of activity, or potentially more than one strain with this activity in the samples (see below). Bacteriocins and bacteriophages can have broad or narrow spectrums of activity (Penadés et al., 2015; Simons et al., 2020). Note that the approach used to evaluate cell-free extracts was only semi-quantitative, as interpretation could be somewhat subjective, particularly for “weak” and “very weak” spots. Size of spots was also influenced by length of storage, with reduced activity over time (data not shown). Nonetheless, general trends could be determined with the screening approach used in this study.

Recovery of inhibiting organisms from enrichment cultures was challenging. Out of 13 enrichment cultures, seven *E. coli* isolates were recovered from cultures derived from raw meat products using a triple agar overlay method (Henning et al., 2015; Table 2). Recovery of inhibitor-producing *E. coli* is consistent with observations for the food-associated bacterial strains, and further supports the finding that *E. coli* may be an important source of inhibition, particularly for growth of *S. sonnei*. Results may be biased by the use of only one of the indicator strains (*S. sonnei*, OLC2340) for the recovery of inhibitory isolates, which were all derived from meat enrichments. *E. coli* is an indicator of fecal matter contamination which could occur during the slaughter process, potentially explaining the association of these organisms with raw meat products (Tallon et al., 2005; Odonkor and Ampofo, 2013; Barco et al., 2015). Failure to recover isolates in six samples may be because the method required recovery of a bacterial strain producing the inhibition. This approach would not work for the recovery of bacteriophage that may have been present in the foods, but not associated with a bacterial host. Cell-free extracts from the isolated *E. coli* strains had similar inhibitory properties to the extracts from the food samples from which they were isolated (Table 2; Supplementary Table S3), particularly to *S. sonnei*, which was the organism used for the recovery of these isolates. However, differences in activity against other indicator strains were observed. For example, OLC3032 was active against *S. flexneri*, unlike in the original enrichment culture where only *S. sonnei* was affected and conversely OLC3028 did not inhibit growth of *S. flexneri*, unlike the original food enrichment culture. This indicates that multiple strains may have been responsible for inhibitory activity in the more complex enrichment cultures. Use of multiple indicator strains, and characterization of additional bacterial isolates may have led to recovery of additional strains.

*S. sonnei* and *S. flexneri* were included as indicator organisms in this study as they are the *Shigella* species most commonly associated with outbreaks in North America (Thompson et al., 2015; Centers for Disease Control and Prevention (CDC), 2016; The et al., 2016). *Shigella sonnei* was most commonly affected by antimicrobial compounds, followed by *S. flexneri* (Figures 3, 5). Previous studies have that shown that *S. flexneri* was more highly affected by organic acids (e.g., by-products from food and food microbiota) than *S. sonnei* (Hentges, 1969; Uyttendaele et al., 2001; Zhang et al., 2011). *S. dysenteriae* was not affected by any of the cell-free extracts, so it is possible this species has a tolerance or resistance to inhibitors such as bacteriocins due to differences in targeted receptors in this species (Alonso et al., 2000). The acidity of the cultures was not likely to be an important factor in the observed growth inhibition. The pH range of *Shigella* growth is between 4.8–9.3 and the range of STEC growth is 4.0–10.0 (Food and Drug Administration, 2011). The pH of the bacterial isolate cell-free extracts (6.5–7.5) and food enrichment extracts (6.0–7.5) was well within these ranges.

Foodborne *Shigella* outbreaks are often associated with plant products (Kozak et al., 2013; Nygren et al., 2013). While enrichment broths derived from plant products were less likely to contain bacteriocins than those derived from meats in this study, this does not mean that recovery of *Shigella* spp. from plant products would not be problematic. *Shigella* contamination of foods is exclusively from human sources, and such contamination would likely also include *E. coli*. Given that human isolates of *E. coli* commonly inhibit *Shigella* spp. (Halbert, 1948; Levine and Tanimoto, 1954), the combination of organisms could impact recovering *Shigella* from the implicated foods. In fact, in a foodborne outbreak associated with baby corn in 2007, Shigella was not recovered from implicated samples despite strong epidemiological evidence, however, samples had high levels of *E. coli* (100–350 cfu/g) indicating potential issues with food hygiene (Lewis et al., 2009).

### Identification of Predicted Bacteriocins and Bacteriophage in Cell-Free Extracts

To gain a better understanding of the mechanism of the inhibition, cell-free extracts were digested with proteolytic enzymes to determine if the inhibitor was impacted, as would be expected for bacteriocins which are proteinaceous compounds (Elayaraja et al., 2014), and extracts were diluted to identify the plaques that would be attributed to bacteriophages (Hockett and Baltrus, 2017; Figure 7). Most of the inhibitors, particularly those affecting *S. sonnei*, were affected by proteolytic enzymes and are likely to be bacteriocins (Figures 3, 5, 7). Notably, there were two *E. coli* strains (OLC1028 and OLC1219) that were predicted to produce both bacteriocins and bacteriophages with antimicrobial activity against *Shigella* spp. (Figure 3). In contrast, STEC inhibition was more commonly associated with bacteriophages (Figures 3, 5). The *Enterobacter* strains that inhibited growth of the O45 and O103 were both predicted to produce bacteriophages (Figure 3). Similarly, the broad spectrum inhibition of STEC observed for sample GTA-1452 was also determined to be associated with bacteriophages. This inhibition could be due to the presence of a bacteriophage that has a wide spectrum of activity, or multiple bacteriophages targeting a few strains (Penadés et al., 2015; Simons et al., 2020). For example, STEC-killing phage can have broad or narrow host ranges (Litt et al., 2018; Mangieri et al., 2020). Interestingly, the inhibition produced by the isolate recovered from the STH-2768 m (OLC3032) was determined to be due to bacteriophages (Table 2) rather than the bacteriocins observed in the original culture (Supplementary Table S3), indicating that there was likely at least one bacteriocin-producing organism in the original culture that was not recovered. The identification of multiple inhibiting organisms within a single enrichment culture is further evidence that these strains may be an important factor in the success of competitive enrichment for food microbiology methods.

Bacteriophages active against STEC have been isolated from numerous zoonotic sources and may be relatively common indicating potential importance for enrichment cultures (Litt et al., 2018; Mangieri et al., 2020; Pinto et al., 2021). Presence of bacteriophages active against target species in enrichment cultures has been shown to reduce relative proportions of target *Salmonella* and *S. sonnei* by over a log indicating their critical impact on food microbiological methods (Muniesa et al., 2005). Bacteriocins would be expected to have similar inhibitory activity in food enrichment cultures. The ability to produce bacteriocins impacting closely related species is a common feature of *E. coli* enabling their survival in competitive environments (Micenková et al., 2016; Cameron et al., 2019). *E. coli*, particularly human isolates, frequently produce colicins, some of which have been shown to specifically target *Shigella* spp. (Halbert and Gravatt, 1949; Levine and Tanimoto, 1954). Only two colicins (colicin Z and J_s_) that specifically target Enteroinvasive *E. coli* (EIEC) and *Shigella* species have been characterized (Micenková et al., 2019). Further analysis of the *E. coli* recovered in this study will be conducted to determine if the strains recovered encode related colicins.

In this study, *S. sonnei* were more susceptible to bacteriocins than *S. flexneri* and STEC. Due to practical considerations, the number of indicator strains used in this study was necessarily limited. It is possible that some of the indicator organisms used had higher resilience to phage and bacteriocins due to biological factors such as presence of immunity proteins, or other defense mechanisms (Rendueles et al., 2022). Use of a larger panel of *Shigella* strains, particularly strains associated with food outbreaks, will be necessary to establish whether *S. sonnei* strains are more susceptible than others. An understanding of the impact of bacteriocins on enrichment dynamics could lead to approaches to mitigating their impact. For example, fermentable sugars have been shown to reduce colicin production by *S. sonnei* (Lavoie et al., 1973). Future studies will target enrichment dynamics associated with bacteriocins in food matrices.

While outside of the scope of the current study, there is currently significant interest in alternatives to antibiotics for the prevention of food contamination with these *Shigella* spp. and STEC, and for the development of therapeutics (Yang et al., 2014; Mangieri et al., 2020; Telhig et al., 2020; Jiang et al., 2022). The bacteria recovered in this study are highly active against pathogenic strains of *Shigella* spp. and STEC, with potential for future use in the production of alternatives to antibiotics.

## Conclusion

Isolation of a foodborne bacterial pathogen is an important component of a food safety investigation that is sometimes difficult to achieve. This study provides evidence that production of antimicrobial compounds such as bacteriocins and bacteriophages by food microbiota is a relatively common occurrence which could impede growth of *Shigella* and STEC in enrichment cultures, reducing effectiveness of microbiological methods aimed at recovering these organisms. Presence of bacteriocin-producing *E. coli* may be an important challenge for the recovery of *Shigella* spp. and presence of bacteriophage may be of greater concern for STEC. Development of new methods should mitigate the potential interference due to inhibitor production by food microbiota. Organisms recovered in this study could be included as interfering organisms in future method validations, leading to a more rigorous assessment of methods aimed at recovery of *Shigella* and STEC from foods.

## Data Availability Statement

The original contributions presented in the study are included in the article/Supplementary Material, and further inquiries can be directed to the corresponding author.

## Author Contributions

CK originally identified bacteriocins as an important inhibitor for *Shigella* spp. TM, CC, AW, and BB conceived and designed the experiments and contributed to writing of the manuscript. TM performed laboratory experiments. TM and CC analyzed the data and wrote the first draft of the manuscript. TM, CC, AM, KS, and BB contributed reagents, materials, and analysis tools. All authors contributed to the article and approved the submitted version.

## Funding

This study has received funding from the Canadian Food Inspection Agency and the Government of Canada interdepartmental Genomic Research Development Initiative (GRDI).

## Conflict of Interest

The authors declare that the research was conducted in the absence of any commercial or financial relationships that could be construed as a potential conflict of interest.

## Publisher’s Note

All claims expressed in this article are solely those of the authors and do not necessarily represent those of their affiliated organizations, or those of the publisher, the editors and the reviewers. Any product that may be evaluated in this article, or claim that may be made by its manufacturer, is not guaranteed or endorsed by the publisher.
